# Surgical management of tuberculum sellae meningioma: Transcranial approach or endoscopic endonasal approach?

**DOI:** 10.3389/fsurg.2022.979940

**Published:** 2022-08-31

**Authors:** Kang Qian, Chuansheng Nie, Wende Zhu, Hongyang Zhao, Fangcheng Zhang, Haijun Wang, Xiaobing Jiang

**Affiliations:** Department of Neurosurgery, Union Hospital, Tongji Medical College, Huazhong University of Science and Technology, Wuhan, China

**Keywords:** tuberculum sellae meningioma, transcranial approach, endoscopic endonasal approach, gross total resection, cerebrospinal fluid leakage

## Abstract

**Background:**

Tuberculum sellae meningioma (TSM), a common benign tumor in the sellae region, usually causes neurological deficits, such as vision impairment, by squeezing the peripheral neurovascular structures. Surgical management is recommended as the optimal strategy for TSM treatment and vision restoration. However, it remains challenging to resect TSM in the traditional transcranial approach (TCA). Recently, the endoscopic endonasal approach (EEA) has emerged as an effective option in skull base surgeries. Besides the effectivity, the advantages and limitations of EEA in TSM surgery remain controversial.

**Object:**

We compared the surgical outcomes and complications between TCA and EEA surgeries to identify the principles in TSM surgical management.

**Methods:**

Retrospective analysis was performed on the patients, who underwent TSM surgery in Wuhan Union Hospital between January 2017 and December 2021. The patients were assigned to TCA or EEA group according to the surgery they experienced. All patients were analyzed with the extent of tumor resection, vision outcome, postoperative complications, and follow-up results.

**Results:**

A total of 112 patients were enrolled in this study, including 78 in TCA group and 34 in EEA group. The mean follow-up was 20.5 months (range 3–36 months). There were no statistically significant differences in patient demographic data, preoperative symptoms, and tumor characteristics between TCA and EEA groups. Both TCA and EEA surgeries are effective in TSM resection with relatively high gross total resection rates (85.9% in TCA vs. 91.2% in EEA, *p* > .05). Meanwhile, EEA surgery has a better outcome in vision restoration or stabilization than TCA surgery (74.6% in TCA vs. 93.1% in EEA, *p* < .05). Whereas EEA surgery causes more occurrences of cerebrospinal fluid (CSF) leakage than TCA surgery (0% in TCA vs. 11.8% in EEA, *p* < .05).

**Conclusion:**

Both TCA and EEA surgeries are effective in TSM resection. EEA surgery has a better outcome in vision restoration or stabilization than TCA surgery, but induces higher risk of CSF leakage. As each approach has unique advantages and limitations, we must take all aspects into consideration, including approach feathers, tumor characteristics, and clinical requirements, to make the optimal choice in TSM surgical management.

## Introduction

Tuberculum sellae meningioma (TSM) is a special type of meningioma located in the suprasellar region and accounts for approximately 5%–10% of all intracranial meningiomas (1). Generally, TSM comprises meningioma arising from tuberculum sellae, limbus sphenoidale, chiasmatic sulcus, and diaphragm sellae (2). Seungjoo et al. demonstrated that 85% of TSMs tend to grow in the midline and usually cause optic nerve/chiasm lateral or superior displacement (3). It was also reported that about 56%–77% of TSMs invade the optic canal, resulting in optic nerve compression (4, 5). Therefore, the most common clinical manifestation of TSM patients is progressive vision impairment. Other clinical manifestations of TSM patient comprise headache, anosmia, seizures, and pituitary dysfunction (6).

The primary goals of TSM surgical management are tumor gross total resection (GTR) and vision restoration. However, it remains challenges in TSM surgical management, since TSMs are anatomical proximity to the vital neurovascular structures, such as optic nerve/chiasm, internal carotid artery (ICA) and its branches, pituitary stalk and hypothalamus (7). When TSM is small, the neurosurgeons can easily separate the tumor from the neurovascular structures along the well-preserved arachnoid interfaces. With TSM growing up, the arachnoid interfaces are broken down and the surrounding neurovascular structures are encased by the tumor. Moreover, the vision impairment and visual field defect are progressively exacerbated, resulting in GTR of TSM and vision restoration becoming much more difficult.

Traditional transcranial approaches (TCAs), including pterional, subfrontal, interhemispheric, and supraorbital craniotomy, are familiar to most neurosurgeons. Nevertheless, in the past decade, endoscopic endonasal approach (EEA) emerged as an effective option for neurosurgeons in skull base surgery (6, 8). Both TCA and EEA have been described in literatures with successful surgical outcome and minimum complication in TSM surgery (9, 10). But there are few studies comparing the surgical outcome and postoperative complication between TCA and EEA in TSM surgical management directly. What are their advantages and limitations? Which principles should be followed in surgical management? These controversies remain to be figured out.

In this study, we retrospectively analyzed the surgical outcome and complication of 112 TSM patients, who experienced TCA or EEA surgery in Wuhan Union Hospital. We also presented our experience in TSM surgical management.

## Methods

This retrospective study enrolled all patients of TSM, who experienced TCA or EEA surgery in Wuhan Union Hospital between January 2017 and December 2021. All of these cases were pathologically confirmed as meningioma (WHO grade I). Meningiomas arising from the clinoid processes, olfactory groove and planum sphenoidale were excluded. The surgical indications included progressive headache, intracranial hypertension sign, and vision impairment. All tumors with base diameter or lateral extension over 3.0 cm were managed with TCA surgery. EEA surgery was performed in the cases of midline tumor with base diameter less than 3.0 cm. Preoperative and postoperative clinical reports of these patients were evaluated, including demographics, clinical manifestations, image data, endocrine functions, ophthalmological assessments, operative records, and complications. Endocrine functions were evaluated in 2–4 weeks postoperatively. Image data, including computed tomography (CT) and magnetic resonance imaging (MRI), was used for preoperative evaluation, surgery assessment, and postoperative outcome analysis. During the follow-up, MRI was performed in 48 h and 3–6 months postoperatively. The tumor size was presented as the largest diameters in all three dimensions (length, width, and height), depending on the preoperative MRI. The volume of tumor was calculated by the formula that tumor volume in cubic centimeters (cm^3^) = (anteroposterior × coronal × craniocaudal)/2. In this formula, the tumor configuration was assumed as a rough sphere. The extent of tumor resection was evaluated according to the operation records and postoperative MRI. We defined GTR as no tumor or capsule remnant on postoperative MRI examination, and subtotal resection (STR) as tumor or capsule remnant.

Traditional TCA surgeries, including pterional, subfrontal, interhemispheric and supraorbital craniotomy, and extended EEA surgery were provided to remove the tumor in this study. All surgeries were performed by senior neurosurgeons in our department.

### Statistical analysis

The data were analyzed by SPSS 26.0. Descriptive statistics were presented as tables and used to analyze patient demographics. Continuous variables were presented as mean values with SDs. Categorical variables were described as percentages. Group comparisons were evaluated by the Student’s *t*-test or Chi-square test. The value of *p* < .05 was regarded as statistically significant difference.

## Results

### Clinical characteristics

A total of 112 TSM patients were enrolled in this study. Among these patients, 78 were performed TCA surgery and assigned to the TCA group, 34 were performed EEA surgery and assigned to the EEA group. The mean follow-up period was 20.5 months (range 3–36 months).

The TCA group is comprised of 30 (38.5%) males and 48 (61.5%) females with a mean age of 50.5 ± 11.7 years. The EEA group is comprised of 12 (35.3%) males and 22 (64.7%) females with a mean age of 52.2 ± 10.1 years. The most common symptom was vision impairment, which was observed in 92 (82.1%) patients, including 63 (80.8%) in TCA group and 29 (85.3%) in EEA group. Headache was presented in 46 (41.1%) patients, including 33 (42.3%) in TCA group and 13 (38.2%) in EEA group. According to the imaging findings, dura tail sign was found in 77 (68.8%) patients, with 55 (70.5%) in TCA group and 22 (64.7%) in EEA group. We also listed the main optic nerve-related vessels and evaluated their relationship with tumor. The results showed 71 (63.4%) patients, including 52 (66.7%) in TCA group and 19 (55.9%) in EEA group, exhibited ICA involvement; 43 (38.4%) patients, including 29 (37.2%) in TCA group and 14 (41.2%) in EEA group, exhibited ophthalmic artery (OA) involvement; 52 (46.4%) patients, including 37 (47.4%) in TCA group and 15 (44.1%) in EEA group, exhibited anterior cerebral artery (ACA) involvement (Supplementary Table S1). Vascular encasement (>180°) was identified in 24 (21.4%) patients, with 18 (23.1%) in TCA group and 6 (17.6%) in EEA group. Optic canal invasion was diagnosed in 67 (59.8%) patients, with 45 (57.7%) in TCA group and 22 (64.7%) in EEA group. Moreover, we compared the degree of optic nerve compression between TCA and EEA groups. The results showed 95 (84.8%) patients, including 65 (83.3%) in TCA group and 30 (88.2%) in EEA group, exhibited optic nerve compression; 61 (54.5%) patients, including 43 (55.1%) in TCA group and 18 (52.9%) in EEA group, exhibited optic nerve displacement; 98 (87.5%) patients, including 68 (87.2%) in TCA group and 30 (88.2%) in EEA group, exhibited optic nerve adhesion; 29 (25.9%) patients, including 22 (28.2%) in TCA group and 7 (20.6%) in EEA group, exhibited optic nerve wrapped by the tumor (Supplementary Table S1). The mean volume of tumor was 11.2 ± 4.8 cm^3^, with 11.5 ± 4.6 cm^3^ in TCA group and 10.7 ± 5.2 cm^3^ in EEA group. There were no statistically significant differences between TCA and EEA groups regarding sex, mean age, preoperative symptom, imaging finding, optic nerve-related vessels involvement, degree of optic nerve compression, and mean tumor volume (*p* > .05) (Table 1).

**Table 1:** Main clinical manifestations of all patients.

	Total (%)	TCA (%)	EEA (%)	*p*-value
No. of patients	112 (100)	78 (69.6)	34 (30.4)	
Mean age (SD)	51.0 (11.2)	50.5 (11.7)	52.2 (10.1)	0.467
Male sex	42 (37.5)	30 (38.5)	12 (35.3)	0.750
Symptoms				
Visual impairment	92 (82.1)	63 (80.8)	29 (85.3)	0.565
Headache	46 (41.1)	33 (42.3)	13 (38.2)	0.687
Image characteristic				
Dural tail sign	77 (68.8)	55 (70.5)	22 (64.7)	0.542
Vascular encasement (>180°)	24 (21.4)	18 (23.1)	6 (17.6)	0.520
Optic canal involvement	67 (59.8)	45 (57.7)	22 (64.7)	0.486
Mean tumor vol. (SD)	11.2 (4.8)	11.5 (4.6)	10.7 (5.2)	0.426

EEA, endoscopic endonasal approach; TCA, transcranial approach; SD, standard deviation.

### Extent of tumor resection

GTR of tumor was achieved in 98 (87.5%) patients, with 67 (85.9%) in TCA group and 31 (91.2%) in EEA group. In addition, we analyzed the removal of tumors invading the optic canal separately. Among the patients of optic canal invaded, 56 (83.6%) patients, including 36 (80.0%) in TCA group and 20 (90.9%) in EEA group, experienced GTR of tumors (Supplementary Table S1). Both TCA and EEA surgeries are effective in TSM resection with relatively high GTR rates. Although there were no statistically significant differences of GTR rates (*p *> .05) between TCA and EEA surgeries in the current study (Table 2). EEA surgery can provide a close and high-definition surgical view for neurosurgeons, which contributes to the identification of anatomical structures and ensures the surgical safety (5, 11, 12).

**Table 2:** Postoperative outcomes and complications.

	Total	TCA (%)	EEA (%)	*p*-value
Gross total resection	98 (87.5)	67 (85.9)	31 (91.2)	0.437
Vision improved or stable	74 (80.4)	47 (74.6)	27 (93.1)	.038
Worsened	18 (19.6)	16 (25.4)	2 (6.9)	.038
CSF leakage	4 (3.6)	0 (0)	4 (11.8)	.002
Meningitis	5 (4.5)	2 (2.6)	3 (8.8)	0.140
Hypopituitarism	15 (13.4)	10 (12.8)	5 (14.7)	0.788
Diabetes insipidus	7 (6.3)	5 (6.4)	2 (5.9)	0.915
Hemorrhage	3 (2.7)	2 (2.6)	1 (2.9)	0.910
Seizures	7 (6.3)	7 (9.0)	0 (0)	.071
Death	0	0	0	

EEA, endoscopic endonasal approach; TCA, transcranial approach; CSF, cerebrospinal fluid.

### Visual outcome

Among the 92 patients with vision impairment, vision restoration or stabilization was reported in 74 (80.4%) patients, including 47 (74.6%) in TCA group and 27 (93.1%) in EEA group. There were statistically significant differences (*p* < .05) between TCA and EEA groups in vision restoration or stabilization rates. On the other hand, 18 (19.6%) patients got worsening vision postoperatively, including 16 (25.4%) in TCA group and 2 (6.9%) in EEA group (Table 2). Overall, EEA surgery has advantages over TCA surgery in vision restoration or stabilization in TSM resection.

### Postoperative complications

In our study, there were 4 (11.8%) patients experienced postoperative cerebrospinal fluid (CSF) leakage in EEA group and 2 (5.9%) of them required a secondary surgery to reconstruct the skull base. Whereas, none of the 78 patients in TCA group experienced CSF leakage. There were statistically significant differences (*p* < .05) between TCA and EEA groups in CSF leakage rates (Table 2). In other words, compared with TCA surgery, EEA surgery may induce a higher risk of CSF leakage in TSM surgical management.

We also observed other complications in the current study, including meningitis (2 in TCA, 3 in EEA), hypopituitarism (10 in TCA, 5 in EEA), diabetes insipidus (5 in TCA, 2 in EEA), hemorrhage (2 in TCA, 1 in EEA), and seizures (7 in TCA, 0 in EEA). No surgery-related death occurred. However, there were no statistically significant differences in these postoperative complications between TCA and EEA groups (*p *> .05) (Table 2).

## Discussion

TSM is a common benign tumor in the sellae region (1). Generally, TSM grows slowly and does not cause any clinical symptoms in early period. With the tumor growing up, TSM squeezes the peripheral anatomical structures, including optic nerve/chiasm, ICA and its branches, pituitary stalk and hypothalamus, causing neurological dysfunctions (13, 14). Impaired visual acuity and visual field are the most common clinical manifestations of TSM. Surgical management is recommended as the optimal strategy for TSM treatment and vision restoration. However, it remains challenge in TSM surgeries, due to the anatomical proximity with vital neurovascular structures in skull base. Traditional TCA surgeries, including pterional, subfrontal, interhemispheric, and supraorbital craniotomy, have been widely applied in TSM resection (9, 10, 14–17). Likewise, with advance in optical technology and improvement in surgical technique, EEA surgery has emerged as an effective option for properly selected TSM patients in the past decade (18, 19).

### Extent of tumor resection

Generally, the extent of tumor resection is an independent predictor of TSM recurrence and has an impact on the surgical outcome. The GTR rate of TSM is about 60%–100% in literatures reviewed (5–7, 20). However, it is difficult to compare the surgical outcomes between different studies directly, due to the lack of uniform criteria (6). In the current study, we use standard criteria to evaluate the degree of tumor resection in 112 patients, with a result of GTR rate 85.9% in TCA group and 91.2% in EEA group.

**Figure 1:**
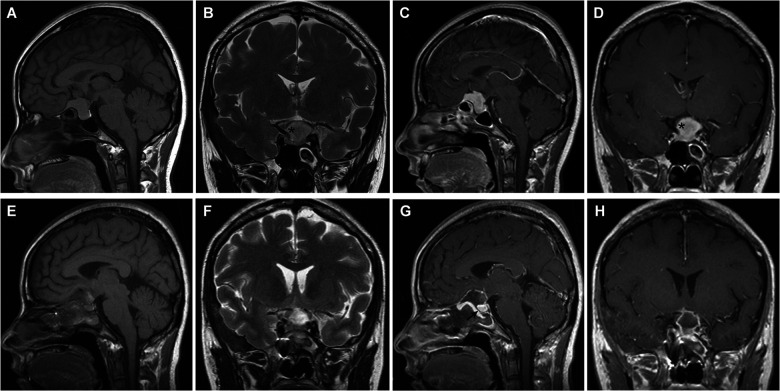
Tuberculum sellae meningioma (WHO grade I). (**A–D**) Preoperative MRI shows an intrasellar and suprasellar tumor with internal carotid artery encasement (>180°) (asterisk). (**E–H**) Postoperative MRI demonstrates gross total resection of the tumor and skull base reconstruction. The optic nerve and pituitary (arrow) were decompressed. Visual acuity and visual field were restored rapidly and pituitary function was preserved after surgery.

**Figure 2:**
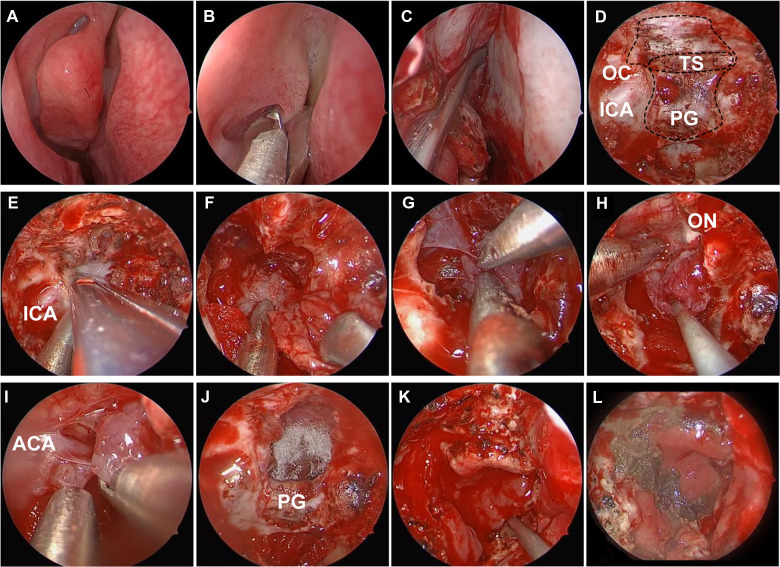
Intraoperative photos of endoscopic endonasal surgery for tuberculum sellae meningioma. (**A**) Nasal mucosa constriction. (**B**) Remove the middle turbinate. (**C**) Vascularized nasoseptal flap separation. (**D**) Expose the anterior fossa dura. (**E**) Enlarge the skull base exposure. (**F**) Intratumor decompression. (**G**) Dissociate the tumor boundary. (**H**) Resect the main part of tumor. (**I**) Dissect the adherent tumor from the anterior cerebral artery complex. (**J**) Gross total resection of tumor. (**K**) Reconstruct the skull base by vascularized nasoseptal flap. (**L**) Probe the nasal 10 days after surgery. ICA, internal carotid artery; PG, pituitary gland; TS, tuberculum sellae; OC, optic canal; ON, optic nerve; ACA, anterior cerebral artery.

In line with recent literatures, multiple tumor characteristics, such as tumor size, optic canal involvement, vascular encasement, intracranial extension, surgery, and radiation history, have an impact on the GTR rate of TSM (5, 13, 20–24). EEA surgery is a better choice for small (<3.0 cm) and midline TSMs, as it provides a close, high-definition surgical view and minimizes the invasion. Herein displays a case of TSM that achieves GTR by EEA surgery (Figures 1, 2). As to the large (>3.0 cm), laterally extensive, firm, or fibrous TSMs, TCA surgery is recommended to perform (9, 10, 13–15, 17, 23, 25, 26). Actually, neurosurgeons prefer to achieve GTR if possible. While in some cases, it may be extremely difficult or even dangerous to achieve that goal. In these cases, STR combined with radiotherapy is advocated to ensure safety and prevent tumor recurrence (27). In addition, the technique of neurosurgeon has a marked impact on the extent of tumor resection (28).

### Visual outcome

Vision impairment is the most common clinical manifestation in the patients harboring TSM (6, 7, 9). Generally, surgical management remains to be the most effective treatment for TSM and contributes to restoring the vision (6). In the current study, there were 92 (82.1%) patients of TSM suffering from vision impairment. Among these cases, 74 (80.4%) patients displayed improved or stable vision postoperatively, including 47 (74.6%) in TCA group and 27 (93.1%) in EEA group. These results reveal that EEA surgery may have tremendous advantages on vision restoration or stabilization in TSM surgical management.

Actually, visual outcomes mainly depend on several factors, including tumor size, degree, and duration of the optic nerves compressed, optic canal involvement, perforating artery protection, and the optic nerve manipulation during tumor removal (29). For instance, subchiasmatic perforating arteries, which play important roles in the optic nerve and chiasm blood supply, are hardly to be identified in the surgical field from above in TCA surgery (24, 30, 31). Conversely, EEA surgery provides a surgical field from below, where the perforating arteries can be observed directly and preserved effectively. EEA surgery causes less disturbances in the blood supply of perforating arteries and minimizes the optic nerve manipulations compared with TCA surgery. These advantages of EEA surgery may contribute to restoring the vision in TSM surgery.

### CSF leakage

CSF leakage is one of the most common postoperative complications in EEA surgery. Abrasion of the skull base and incision of the dura from below make EEA surgery more prone to CSF leakage than TCA surgery (1, 7, 32–34). In our study, 4 (11.8%) cases in EEA group experienced CSF leakage. Meanwhile, the meningitis risk was increased in line with CSF leakage. The CSF leakage and meningitis may prolong the time of hospitalization, enhance the cost of patients, or even lead to death. Autologous thigh broad fascia and vascularized nasoseptal flap are recommended to reconstruct the skull base (31, 35) (Figures 2K, L). Moreover, it is necessary to perform continuous lumbar drainage and apply antibiotics, if CSF leakage occurs (36). Recently, with the surgical technique progressing, the occurrence of CSF leakage keeps decreasing (6).

## Conclusion

Both TCA and EEA surgeries are effective in TSM resection. Meanwhile, EEA surgery acquires a better outcome in vision restoration or stabilization than TCA surgery. Although EEA surgery induces higher risk of CSF leakage, the adverse effect is declining with the surgical technique progressing.

EEA surgery has been recommended as an effective option for properly selected TSM patients, since it offers several advantages in TSM surgical management, including (i) EEA surgery offers a close and high-definition surgical view, which contributes to identifying the anatomical structures clearly and ensures surgical safety. (ii) EEA surgery provides better protection for the small perforating arteries, which supply the optic apparatus from below. (iii) EEA surgery reduces the retraction of brain and cranial nerves, preserves the neurological functions better. (iv) EEA surgery is more effective to perform tumor devascularization before resection. (v) EEA surgery leads to less invasion, faster recovery, and better cosmetic results. However, EEA surgery also has limitations compared to TCA surgery, such as (i) EEA surgery is unavailable to resect large tumors, especially those extend laterally. (ii) EEA surgery is difficult to remove firm or fibrous tumors. (iii) EEA surgery induces higher risk of CSF leakage than TCA surgery.

As each approach has unique advantages and limitations, we must take all aspects into consideration, including approach feathers, tumor characteristics and clinical requirements, to make the optimal choice in TSM surgical management.

## Data Availability

The original contributions presented in the study are included in the article/**Supplementary Material**, further inquiries can be directed to the corresponding author/s.
