# Supplementary material for: Surgical management of tuberculum sellae meningioma: Transcranial approach or endoscopic endonasal approach?
Source: Front Surg. 2022 Aug 31;9:979940. doi: 10.3389/fsurg.2022.979940 (PMC9470762; doi:10.3389/fsurg.2022.979940)
Supplement: Supplementary file 1 [file Table_3_v1.docx]

| **Supplementary Table 1 \|** Characteristics of the tumors. | | | | |
| --- | --- | --- | --- | --- |
|  | **Total (%)** | **TCA (%)** | **EEA (%)** | **p value** |
| **Optic canal involvement** | 67（59.8） | 45（57.7） | 22（64.7） | 0.486 |
| **Removal of tumors invading the optic canal** | 56（83.6） | 36（80.0） | 20（90.9） | 0.258 |
| **Degree of optic nerve compression** |  | | | |
| **Compression** | 95（84.8） | 65（83.3） | 30（88.2） | 0.506 |
| **Displacement** | 61（54.5） | 43（55.1） | 18（52.9） | 0.831 |
| **Adhesion** | 98（87.5） | 68（87.2） | 30（88.2） | 0.877 |
| **Wrapping** | 29（25.9） | 22（28.2） | 7（20.6） | 0.397 |
| **Optic nerve related vessels involvement** |  | | | |
| **ICA** | 71（63.4） | 52（66.7） | 19（55.9） | 0.276 |
| **OA** | 43（38.4） | 29（37.2） | 14（41.2） | 0.689 |
| **ACA** | 52（46.4） | 37（47.4） | 15（44.1） | 0.746 |
| *EEA, endoscopic endonasal approaches; TCA, transcranial approaches; ICA, internal carotid artery; OA, ophthalmic artery; ACA, anterior cerebral artery.* | | | | |
